# Associations between Single-Nucleotide Polymorphisms in Corticotropin-Releasing Hormone-Related Genes and Irritable Bowel Syndrome

**DOI:** 10.1371/journal.pone.0149322

**Published:** 2016-02-16

**Authors:** Ayaka Sasaki, Naoko Sato, Naoki Suzuki, Michiko Kano, Yukari Tanaka, Motoyori Kanazawa, Masashi Aoki, Shin Fukudo

**Affiliations:** 1 Department of Behavioral Medicine, Tohoku University Graduate School of Medicine, Sendai, Japan; 2 Department of Neurology, Tohoku University Graduate School of Medicine, Sendai, Japan; 3 Frontier Research Institute for Interdisciplinary Sciences, Tohoku University, Sendai, Japan; Medical University of Gdańsk, POLAND

## Abstract

Irritable bowel syndrome (IBS) is a common functional disorder with distinct features of stress-related pathophysiology. A key mediator of the stress response is corticotropin-releasing hormone (CRH). Although some candidate genes have been identified in stress-related disorders, few studies have examined CRH-related gene polymorphisms. Therefore, we tested our hypothesis that single-nucleotide polymorphisms (SNPs) in CRH-related genes influence the features of IBS. Methods: In total, 253 individuals (123 men and 130 women) participated in this study. They comprised 111 IBS individuals and 142 healthy controls. The SNP genotypes in CRH (*rs28364015* and *rs6472258*) and CRH-binding protein (CRH-BP) (*rs10474485*) were determined by direct sequencing and real-time polymerase chain reaction. The emotional states of the subjects were evaluated using the State-Trait Anxiety Inventory, Perceived Stress Scale, and the Self-rating Depression Scale. Results: Direct sequencing of the *rs28364015* SNP of CRH revealed no genetic variation among the study subjects. There was no difference in the genotype distributions and allele frequencies of *rs6472258* and *rs10474485* between IBS individuals and controls. However, IBS subjects with diarrhea symptoms without the *rs10474485* A allele showed a significantly higher emotional state score than carriers. Conclusions: These results suggest that the CRH and CRH-BP genes have no direct effect on IBS status. However, the CRH-BP SNP *rs10474485* has some effect on IBS-related emotional abnormalities and resistance to psychosocial stress.

## Introduction

Irritable bowel syndrome (IBS) is a widespread functional disorder of the lower gastrointestinal tract that is primarily characterized by abdominal pain and altered bowel habits (diarrhea and/or constipation) [1]. IBS is associated with impaired quality of life and excessive use of health care resources and has long been considered to be a stress-related disorder [2, 3]. Co-morbidity of psychological disorder and somatic co-morbidities have been reported in IBS [4, 5]. IBS patients reported more anxiety, depression, and perceived stress than healthy people [4]. The impact of stress and psychosocial factors on predisposition to IBS symptoms and their precipitation and maintenance highlights the importance of taking into consideration the effect of stress on the underlying molecular mechanisms [6, 7].

Some hypothalamic-pituitary-adrenal (HPA) axis-related genetic variation [8], including 11β-hydroxysteroid dehydrogenase type 1[9], glucocorticoid receptor [10], and mineralocorticoid receptor [11] gene polymorphisms, is known to be associated with the stress response. Corticotropin-releasing hormone (CRH), encoded by the CRH gene, is a key integrator of the stress response [12–14]. The response to stress in mammals is mediated through endocrine, autonomic, and behavioral systems via secretion of CRH by the paraventricular nucleus of the hypothalamus [15, 16]. This system is critical for survival but its chronic over-activity can lead to stress-related pathologies [17–19]. In this way, dysregulation of this system has been linked to a variety of stress-related disorders, including depression, post-traumatic stress disorder, suicidality, panic disorder, and alcohol consumption [20–27]. Components of the highly interrelated system of the HPA axis include not only CRH but also CRH receptors (CRH-R1/CRH-R2) and CRH-binding protein (CRH-BP) [14]. CRH-BP represents a passive ligand trap that binds to and neutralizes CRH, thereby terminating its biological actions, in contrast to its active receptor that initiates signal transduction on binding [28]. Furthermore, CRH-related peptides are expressed not only in the brain but also within the colon, where they activate enteric, endocrine, and immune cells and may be involved in colonic manifestations of IBS [1].

Recently, some candidate gene studies have identified an association between gene polymorphisms and IBS [29–32]. Most of the studies investigated the association of IBS with genetic variations in the serotonin (5-hydroxytryptamine) signaling system, immune system, or adhesion molecules [29–31]. Single-nucleotide polymorphisms (SNPs) in interleukin-10 (IL-10) and hydroxytryptamine receptor 2A were associated with diarrhea-predominant IBS in female patients [30]. IL-6, cadherin-1, and toll-like receptor-9 genes were associated with post-infectious IBS [29]. However, only a few gene studies have found positive evidence of CRH-related gene polymorphisms associated with IBS [32]. Polymorphisms in CRH-related peptide CRH-R1 and CRH-R1 haplotypes were suggested to moderate IBS and IBS bowel patterns in a previous study [32]. Although CRH-related peptides are clearly important in IBS, no study has clarified the role of gene polymorphisms in CRH and CRH-BP in IBS. Earlier studies suggested an association of CRH-BP gene polymorphisms with the antidepressant treatment response, major depressive disorder, and suicidal behavior in schizophrenia [33–35]. In rhesus macaques, a polymorphism in the CRH promoter region increases the risk of disorder in stress-exposed individuals [22].

Thus, the aim of this study was to investigate the associations between genetic variants of the CRH system and IBS. We had the following two hypotheses: 1) SNPs in the CRH and CRH-BP genes are different between IBS patients and healthy people; and 2) these selected SNPs in the CRH and CRH-BP genes influence the IBS endophenotype (bowel movement patterns and psychological abnormalities).

## Materials and Methods

### Subjects

The characteristics of the subjects are shown in **Table 1**. In total, 253 volunteers (130 women and 123 men) aged 22.0 ± 2.2 years (mean ± standard deviation [SD]) participated in the current study. They were all Japanese individuals recruited from universities in Sendai, Japan. IBS patients were diagnosed according to the Rome III criteria [36]. IBS patients were classified by their predominant bowel pattern: constipation, diarrhea, or mixed. There was no significant difference in age, sex ratio, and IBS status between the groups. This study was approved by the Ethics Committee of Tohoku University Graduate School of Medicine. All participants gave their written informed consent.

**Table 1:** Sample Characteristics.

		IBS (n = 111)	Control (n = 142)
**Male**		47	76
**Female**		64	66
**Age, years [SD]**		21.9 [2.0]	22.0 [2.3]
**IBS subtype**			
	**Constipation**	33	0
	**Diarrhea**	46	0
	**Mixed**	32	0

Abbreviations: IBS, irritable bowel syndrome; SD, standard deviation.

Among the 253 participants, 190 were included in a study of the polymorphisms of the 5-hydroxytryptamine transporter [37] and CRH-R1 polymorphic region [32]. However, those genes were completely different from the genes targeted in this study. The hypotheses in this study and the previous studies are different. The previous analysis does not impact on this study.

### Psychometric Tests

The emotional states of the subjects were rated using the Self-rating Depression Scale (SDS) [38, 39], Perceived Stress Scale (PSS) [40, 41], and State-Trait Anxiety Inventory (STAI) [42, 43]. The SDS is a rating of affective, psychological, and somatic symptoms associated with depression, the PSS measures the perception of stress, and the STAI is a rating of two types of anxiety: state anxiety and trait anxiety.

### Genotyping

Whole blood (10mL) was sampled from the median cubital vein of all subjects. DNA was extracted from the white blood cells using a standard protocol [32]. SNPs in CRH (*rs28364015* and *rs6472258*) [22, 33] and CRH-BP (*rs10474485*) [33] were selected by considering previous reports, and minor allele frequencies were determined from a database [NCBI: http://www.ncbi.nlm.nih.gov/snp/]. CRH is encoded by the CRH gene, which is located on chromosome 8 and spans 2.34 kb. *rs28364015* and *rs6472258* are also located on chromosome 8 (position 67253453 and 67259033, respectively). CRH *rs28364015* is a 5′ untranslated region variant, and *rs6472258* is an intergenic variant. Further, CRH-BP is encoded by the CRH-BP gene, which is located on chromosome 5 and spans 16.76 kb. CRH-BP *rs10474485* is located on chromosome 5: (position 76306609). *rs10474485* is also intronic and is a non-coding transcript variant.

The genotypes of the CRH and CRH-BP polymorphisms were determined by real-time polymerase chain reaction (PCR) (TaqMan assay). DNA samples from 40 participants were amplified by direct PCR to determine the positive controls. A 50-μL PCR reaction consisted of 0.2 μM of each primer, 1.25 U Primer STAR HS DNA polymerase, 200 μM deoxynucleoside triphosphate, and 1× Prime STAR buffer. After initial denaturation at 94°C for 4 min, amplification was performed using 35 cycles at 94°C for 1 min (denaturation), 60°C for 1 min (annealing), and 72°C for 1.5 min (elongation), followed by a final elongation step at 72°C for 7 min. Then, the amplified PCR products were purified from a 2% agarose gel using a QIAquick Gel Extraction Kit (QIAGEN, Hilden, Germany). Amplimers were directly sequenced using an ABI PRISM dRhodamine^TM^ Terminator Cycle Sequencing Ready Reaction Kit (PE Applied Biosystems, Foster City, CA), and excess dye terminators were removed using CENTRI-SEP columns (Princeton Separations, Adelphia, NJ). Automated sequencing was performed on an ABI 3130 Genetic Analyzer (PE Applied Biosystems). All procedures were carried out according to the instructions of the manufacturers.

Forward and reverse primers were used to sequence the PCR products. Using Primer3 Input version 0.4.0 (http://frodo.wi.mit.edu/primer3/), two CRH SNP (*rs28364015* and *rs6472258*) and one CRH-BP SNP (*rs10474485*) oligonucleotide primer sets were designed. The forward primers were 5′-GCA GAA AGA TGG TGG GAC TC-3′ (*rs28364015*), 5′-AGG AGA ATC GCT TGA ACC TG-3′ (*rs6472258*), and 5′-GCA CCC AAA AGA GAG TTG TG-3′ (*rs10474485*). The reverse primers were 5′-TCT CTT GAC AGC TCG ATT GC-3′ (*rs28364015*), 5′-CTG GAT TGA ATT CCC TGT CC-3′ (*rs6472258*), and 5′-GGA GAG TCA ACA GGG GAA TTG-3′ (*rs10474485*).

Similarly, *rs6472258* and *rs10474485* TaqMan probe sets were designed, and the participants were genotyped by real-time PCR. However, the TaqMan assay failed for *rs28364015* because of the frequency of the C allele (0%). The PCR reactions consisted of 200 nM of each primer, 200 nM fluorogenic probe, 1× SsoAdvanced Probes Supermix, and distilled water (up to 10 μL). Automated sequencing was performed on a CFX 96^TM^ Real-Time PCR Detection System (Bio-Rad Laboratories, Inc., Tokyo, Japan).

### Statistical Analysis

We used Haploview [44] to confirm the Hardy-Weinberg equilibrium. The effects of the genotypes or alleles of each SNP were compared between the IBS patients and controls using the χ^2^ test and Odds Ratio (OR) (95% confidence interval [CI]). Two-way analysis of variance with a post hoc test was performed to determine the association between the SNPs and the psychometric tests. Results are expressed as the mean ± SD. We considered p < 0.05 to be statistically significant, whereas a p-value < 0.1 was considered to be tendentially but not statistically, significant. Statistical analyses were performed using SPSS Statistic 22.0 (IBM, Inc., New York, NY).

## Results

The genotype frequencies of the selected CRH and CRH-BP gene variations in the IBS patients and controls are shown in Table 2. There was no significant difference in the genotype frequencies of *rs6472258* (p = 0.990, χ^2^ test) and *rs10474485* (p = 0.969, χ^2^ test) between the patients and controls. Similarly, there was no significant difference in the allele frequencies of the *rs6472258* T allele (p = 0.675, χ^2^ test) or G allele (p = 0.890, χ^2^ test), and *rs10474485* C allele (p = 0.816, χ^2^ test) or A allele (p = 0.986, χ^2^ test) between the IBS patients and controls. Moreover, the results of the additional analysis (OR [95% CI]) in *rs6472258*, alleles are not associated with IBS, with an OR of 1.036 (95% CI, 0.629–1.71) for G allele and 1.015 (95% CI, 0.524–1.97) for T allele. Similarly, the allelic OR (95% CI) of *rs10474485* were 1.000 (95% CI, 0.976–1.025) for A allele and 0.914 (95% CI, 0.427–1.956). There was no difference in statistical results between genotype or allele frequency and OR.

**Table 2:** Genotype Frequencies in the IBS Patients and Controls.

dbSNP rs#	Genotype	IBS (%)		Control (%)		p value
		Male (n = 47)	Female (n = 64)	Male (n = 76)	Female (n = 66)	IBS vs. control
***rs6472258***	**GG**	9 (8.1)	10 (9.0)	15 (10.6)	9 (6.3)	0.990
	**GT**	12 (10.8)	20 (18.0)	21 (14.8)	19 (13.4)	
	**TT**	26 (23.4)	34 (30.6)	40 (28.2)	38 (26.8)	
***rs10474485***	**AA**	7 (6.3)	6 (5.4)	7 (4.9)	11 (7.7)	0.969
	**AC**	16 (14.4)	17 (15.3)	21 (14.8)	20 (14.1)	
	**CC**	24 (21.6)	41 (36.9)	48 (33.8)	35 (24.6)	

Abbreviations: dbSNP, Single Nucleotide Polymorphism Database; IBS, irritable bowel syndrome.

The psychometric scores of the IBS patients and controls are shown in Table 3. For the SDS, the score of the IBS patients was higher than that of the controls (p = 0.016). In addition, for the PSS, the score of the IBS patients was higher than that of the controls (p = 0.028). The STAI score was not significantly different between the two groups (State, p = 0.111; Trait, p = 0.115).

**Table 3:** Psychometric Scores.

	IBS			Control			p value
Psychometric score, mean [SD]	Male (n = 47)	Female (n = 64)	Total (n = 111)	Male (n = 76)	Female (n = 66)	Total (n = 142)	IBS vs. control
**SDS**	40.2 [7.4]	42.8 [6.9]	41.7 [7.2]	37.9 [7.4]	40.8 [9.5]	39.2 [8.6]	0.016
**PSS**	27.4 [8.5]	29.6 [7.9]	28.7 [8.2]	24.4 [9.3]	28.2 [9.3]	26.1 [9.5]	0.028
**STAI (State)**	47.1 [9.0]	44.8 [9.6]	45.8 [9.4]	42.6 [7.7]	45.3 [11.8]	43.9 [9.9]	0.111
**STAI (Trait)**	47.8 [11.0]	49.5 [10.0]	48.8 [10.4]	46.1 [9.2]	47.2 [12.7]	46.6 [10.9]	0.115

Abbreviations: IBS, irritable bowel syndrome; SD, standard deviation; SDS, Self-rating Depression Scale; PSS, Perceived Stress Scale; STAI, State-Trait Anxiety Inventory.

In the analysis of all subjects (IBS patients + controls), *rs10474485* A allele non-carriers showed a higher PSS score than carriers (F[1, 507] = 6.459, p = 0.012). There was a significant group (IBS vs. control) effect with the SDS (p = 0.014) and PSS (p = 0.030) scores. The mean (± SD) PSS score of A allele carriers was 25.56 (± 8.359), whereas that of non-carriers was 28.48 (± 9.303). The other psychometric scores were not significantly different (SDS, p = 0.112; State, p = 0.140; Trait, p = 0.143). In contrast, we analyzed the associations between *rs6472258* (genotype and allele) and the psychometric scores, but there was no significant difference.

**Fig 1:**
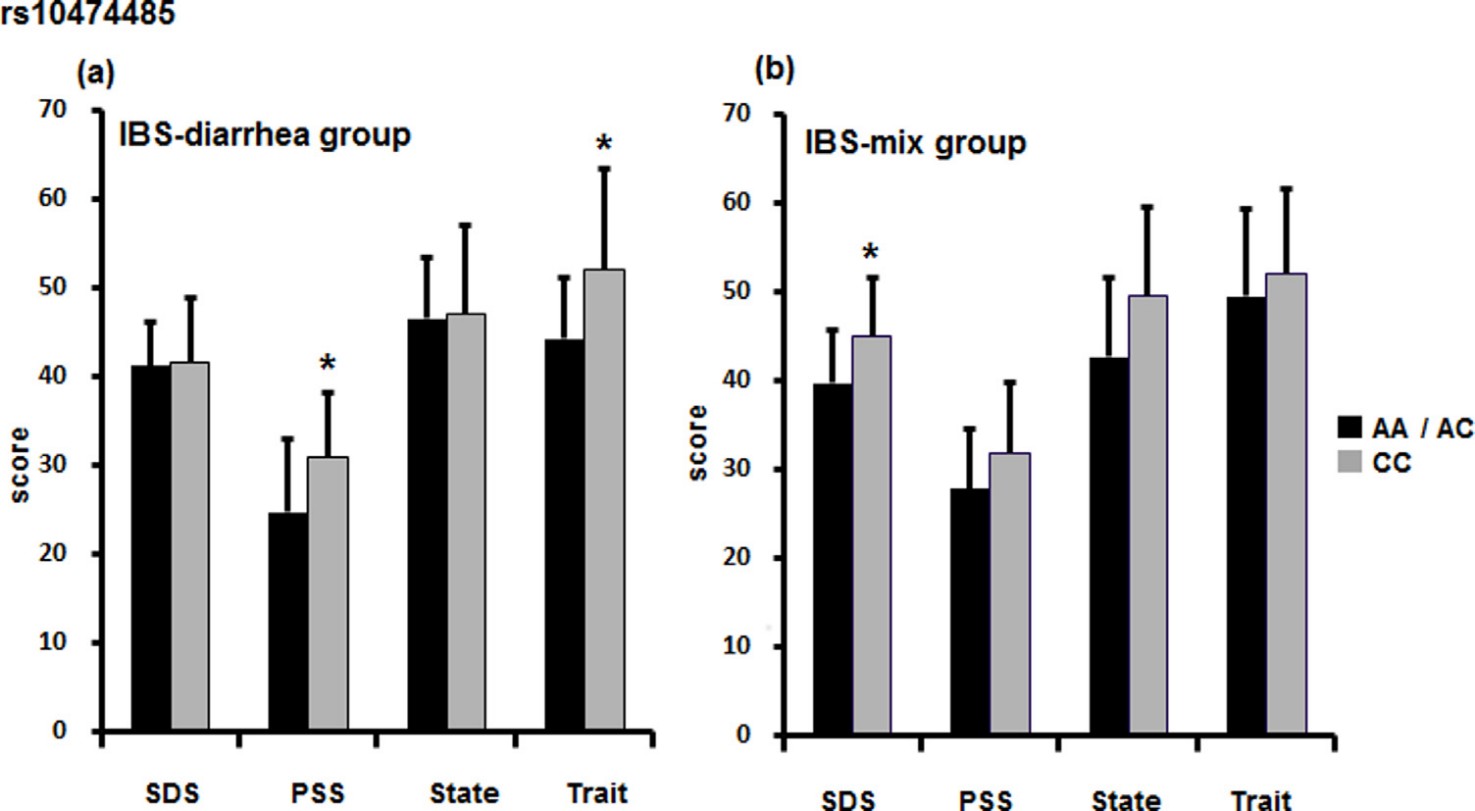
IBS subtype and CRH-BP SNP. We analyzed the associations between the SNPs and psychometric scores according to IBS subtype. (a) In individuals with diarrhea-type IBS, *rs10474485* A allele non-carriers showed higher scores than carriers. There were significant differences in the PSS (p = 0.018) and Trait (p = 0.017) scores. (b) In addition, in the IBS group with mixed symptoms, a significant difference was observed in the SDS score (p = 0.030). There was no significant difference in the constipation group. *p *<* 0.05.

Next, we analyzed IBS symptoms. In individuals with diarrhea symptoms, there was a significant difference in the PSS (F[1, 363] = 6.186, p = 0.018) and Trait (F[1, 589] = 6.192, p = 0.017) scores between *rs10474485* A allele non-carriers and carriers (Fig 1A). The mean (± SD) PSS scores of A allele carriers vs. non-carriers were 24.75 (± 8.234) vs. 30.96 (± 7.252), and the mean Trait scores were 44.19 (± 6.804) vs. 52.09 (± 11.329), respectively. Also, in those with mixed symptoms, the mean (± SD) SDS score of *rs10474485* A allele carriers was 39.75 (± 5.802), whereas that of non-carriers was 45.00 (± 6.585). This difference was significant (F[1, 207] = 5.193, p = 0.030) (Fig 1B). There was no significant difference for those with constipation symptoms. Moreover, the associations between *rs6472258* (genotype and allele) and IBS subtypes were not significantly different.

**Fig 2:**
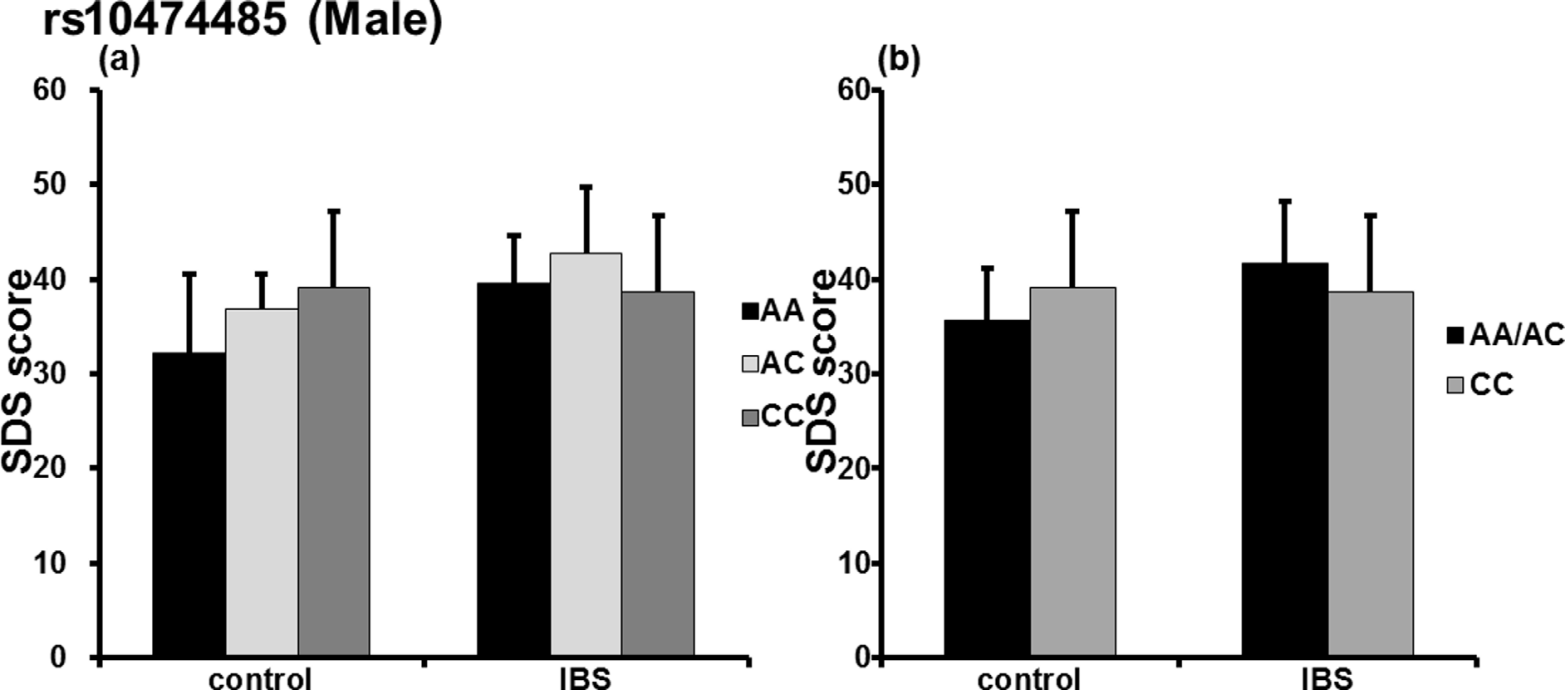
Self-rating Depression Scale and the CRH-BP SNP. We analyzed associations between the selected SNP (*rs10474485*) and psychometric scores according to sex. (a) In male subjects, a significant group (IBS/control) × rs10474485 genotype interaction (p = 0.045) was observed. (b) Further, there was a significant group (IBS/control) × *rs10474485* A allele interaction (p = 0.017). *p *<* 0.05.

**Fig 3:**
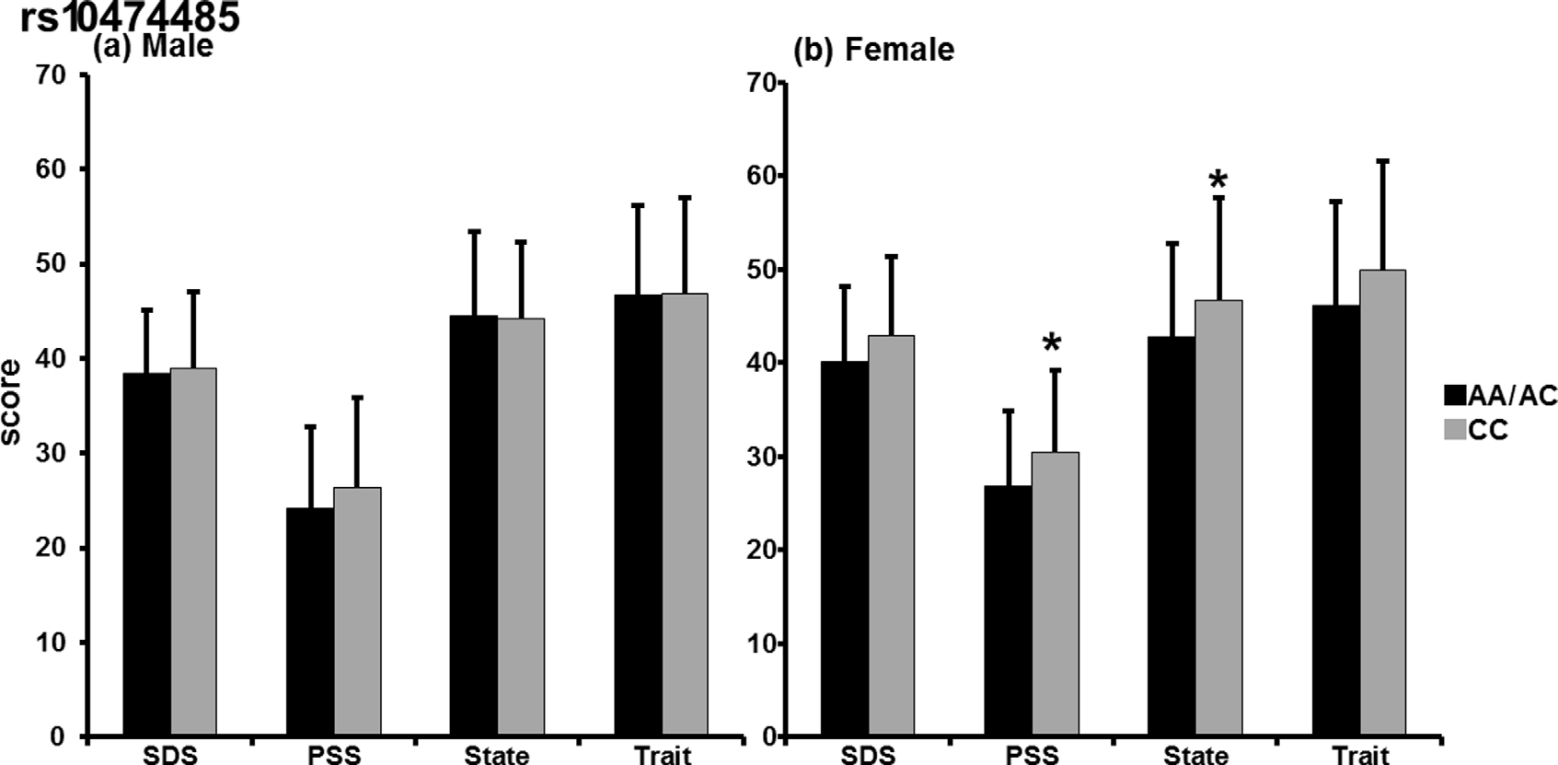
Sex differences in psychometric scores according to the CRH-BP SNP. (a) In analysis of male subjects (IBS + control), there was no significant difference (SDS, p = 0.864; PSS, p = 0.177; State, p = 0.979; Trait; p = 0.976). (b) In contrast, female A allele non-carriers of *rs10474485* showed significantly higher PSS (p = 0.028) and State of STAI (p = 0.035) scores and tended to have higher SDS (p = 0.083) and Trait of STAI (p = 0.078) scores than carriers. *p < 0.05.

In addition, in analysis of the male subjects alone, significant group (IBS/control) × *rs10474485* genotype (F[2, 167] = 3.180, p = 0.045) (Fig 2A) and group (IBS/control) × *rs10474485* A allele (F[1, 309] = 5.821, p = 0.017) (Fig 2B) interaction were observed in the SDS score. However, there was no difference in the emotional scores of the male subjects with respect to the A allele (Fig 3A). In the female subjects, significant differences were observed in the PSS (F[1,360] = 4.931, p = 0.028) and State (F[1, 520] = 4.557, p = 0.035) scores with respect to the *rs10474485* A allele (Fig 3B). The mean (± SD) PSS score of female A allele carriers was 26.87 (± 7.983), whereas that of non-carriers was 30.42 (± 8.843). The mean (± SD) State score of A allele carriers was 42.74 (± 10.091), whereas that of non-carriers was 46.72 (± 10.962). Analyses of *rs28364015* did not indicate a significant difference in males or females.

As for the CRH SNP *rs6472258*, there was a nonsignificant trend for a difference in the SDS score between G allele carriers and non-carriers (all subjects, F[1, 244] = 3.837, p = 0.051; male subjects, F[1, 171] = 3.180, p = 0.077). In the present study, all subjects (n = 40) whose DNA was analyzed with direct sequencing had the *rs28364015* TT genotype and no subjects had the C allele. Therefore, we excluded *rs28364015* from the analysis of the real-time PCR data.

## Discussion

In the current study, we analyzed CRH-related peptide genetic variations in controls and patients with IBS, which is one of the representative disorders with stress-related pathophysiology. On the basis of previous studies and reports of the sexually dimorphic expression of CRH-related genes [45–48], sex differences would be expected in our study. Indeed, in the present study, the CRH-BP SNP *rs10474485* had a significant effect on the PSS score, especially in female subjects. In addition, there was a significant gene × group interaction in the SDS score of males. It has been suggested that sex differences are important when considering genetic influences on IBS. Some previous studies suggested that the functions of hormone and genetic variations were connected to sex [45–49]. For example, a role for 5-hydroxytryptamine receptor 2B genotypes in impulsivity was shown in male subjects [45], and transcriptional regulation of the human CRH-BP gene promoter by estrogen receptors was reported [49]. Similarly, it is thought that the association between differences in sex hormones and gene polymorphisms are important. As CRH plays a dominant role in the HPA axis, the effect of sex differences on hormone and hypothalamic function must be investigated. For the CRH SNP *rs6472258*, a tendency for a difference in the SDS score between G allele carriers and non-carriers was found, which may have influenced the differences in the SDS score in male subjects. Significant results might have been observed with more subjects.

Some studies have suggested that CRH-related gene variations are not associated with IBS *per se*, but that the IBS subgroup or patients’ comorbid anxiety is associated with polymorphisms [50–52]. IBS subjects with diarrhea showed a higher response (namely, more inhibition) than healthy controls to a CRH receptor antagonist [53]. Therefore, IBS subjects with diarrhea might be more responsive to CRH or CRH-related peptides than healthy controls. Here, the IBS subjects with diarrhea symptoms (diarrhea or a mix) showed significant differences in some emotion states according to the presence of the *rs10474485* SNP. It is suggested that these CRH-BP polymorphisms might more strongly influence IBS patients with diarrhea symptoms. CRH is a major mediator to activate HPA axis in stress response. In contrast, CRH-BP reduces the effect of CRH [28]. If *rs10474485* A allele make the CRH-BP effects increase, CRH function may be more interfered with. Thereby, it is suggested that HPA axis activity attenuated, and stress response decreased. As a result, it is thought that psychometric scores might decrease when individuals have A allele.

In the present study, the CRH G and CRH-BP A alleles were minor alleles. According to previous studies of stress-related disorders, CRH-related gene-specific polymorphisms appeared to protect against adult depressive symptoms [54, 55]. Subjects without the minor alleles of the selected SNPs showed higher psychometric scores than minor allele carriers. CRH exaggerates various stress responses, such as anxiety. Conversely, human CRH-BP binds to CRH and prevent activation of CRH receptors, thereby, reduce high activity with CRH [56]. In rodents, CRH-BP cells are concentrated within a hypothalamic circuit involved in mediating neuroendocrine and autonomic responses to stress [57]. CRH-BP expression downregulates the HPA axis by interfering with CRH, and the stress responses converge [28].

Here, CRH-BP *rs10474485* was not directly related to IBS. However, the selected CRH-BP polymorphism was found to be in linkage disequilibrium with the CRH-BP SNPs *rs10055255* (r^2^ = 0.410, D′ = 0.928) and *rs1875999* (r^2^ = 0.410, D′ = 0.928) in the 1000 Genomes Project of Japanese individuals in Tokyo [the 1000 Genomes Browsers: http://browser.1000genomes.org/]. Homozygosity of the *rs10055255* minor allele (TT) was associated with fewer incidences of post-traumatic stress disorder and depressive symptoms in post-intensive care units [58]. Under a condition of stress manipulation, individuals homozygous for the *rs10055255* minor allele (TT) were noted to show high stress-induced alcohol craving, tension, and negative mood compared with their pre-stress condition. During the neutral imagery condition, there was no effect, but the post-stress rating was lower than that of pre-stress [59]. Thus, this SNP (*rs10055255*) may only show the differential effects of stress responses under a stressful situation. However, because our study was not conducted under stress conditions, the *rs10055255* SNP was not selected. Nonetheless, it is suggested that CRH-BP polymorphisms influence stress responses during stress manipulation. In addition, the *rs1875999* major allele (T) was more common in unipolar patients [60], and its minor allele (C) was associated with both heroin and cocaine addiction [61]. At present, the functionality of *rs1875999* is unknown and the directionality of the role of the alleles in the stress response is not consistent. Accordingly, we did not select this SNP in this study. However, it is thought that this SNP has an inhibitory role in the development of stress-related neuropsychiatric disorders. Therefore, it is conceivable that a functional increase may occur in the CRH-BP produced by the CRH-BP *rs10474485* minor allele and that the activity of the HPA axis may decrease. In other words, there may be protective effects of minor CRH/CRH-BP gene variants on the stress response.

Although identified in the rhesus monkey [22], the genetic variant of the *rs28364015* SNP has not been identified in the Japanese population. The present study is the first to try to identify this polymorphism in Japanese individuals. However, the results were negative. Regardless of the limited number of subjects (40 individuals), the possibility of finding this variation is considered to be very low.

There are some limitations to this study. First, we did not have a sufficient number of samples to investigate SNPs. A future direction would be to collect more samples and to explore more SNPs in CRH, CRH-BP, CRH-R1, and CRH-R2. Moreover, because the average age of all subjects was low (22.0 ± 2.2 years), it is thought that the present data could not be extrapolated to the entire IBS population. However, a meta-analysis study showed that there was not significant difference in the prevalence of IBS between different age bands [62]. Therefore, it is thought that the subjects used in the present study were representative as an IBS group. Second, IBS severity was not explored. In the future, the association between disease severity and polymorphisms should be explored. Third, the *rs6472258* and *rs10474485* SNPs selected in this study are intronic. The *rs6472258* SNP is located upstream of CRH gene exon 1. Similarly, *rs10474485* is located downstream of CRH-BP gene exon 7 (Ensembl Genome Browser: http://www.asia.ensembl.org/). The effects of these areas on the transcriptional activity and protein expression of the CRH/CRH-BP genes are not clear. However, in a previous study, CRH receptor-1 SNPs located in the intron around exon 2 were associated with IBS [32]. Therefore, there may be a possibility that intronic SNPs can regulate the function of the CRH/CRH-BP genes. In any future study, the role of CRH genes in the endophenotype of IBS should be explored.

In conclusion, our study suggests that CRH-BP gene polymorphisms had some effect on IBS-related psychological abnormalities. In contrast, we could not detect the associations between the CRH/CRH-BP genes and IBS status per se. Further evidence for a general role of CRH and CRH-BP gene polymorphisms in stress-related disorders is required.
